# Sleep Quality and Cognitive Impairments in Children and Adolescents with Post Traumatic Stress Disorder and/or Depressive Symptoms

**DOI:** 10.3390/jcm14031010

**Published:** 2025-02-05

**Authors:** Mauricio Barrera-Valencia, Liliana Calderon-Delgado, Ana Adan

**Affiliations:** 1 Cognitive Research Group, Department of Psychology, University of Antioquia, Calle 67 No. 53-108, Medellín 050010, Colombia; cliliana7@gmail.com; 2 Philosophical Faculty, Hradec Králové University, Náměstí Svobody 331, 500 02 Hradec Králové, Czech Republic; 3 Department of Clinical Psychology and Psychobiology, School of Psychology, University of Barcelona, Passeig de la Vall d’Hebrón 171, 08035 Barcelona, Spain; aadan@ub.edu; 4 Institute of Neurosciences, University of Barcelona, 08035 Barcelona, Spain

**Keywords:** sleep quality, child and adolescent PTSD, depression, neurocognitive alteration

## Abstract

**Background/Objectives**: Sleep and cognitive alterations are common symptoms associated with child Post-traumatic stress disorder (PTSD) and depression (DEP). This study aims to investigate the relationship between sleep disturbances and cognitive alterations in PTSD and DEP. **Methods**: Using a quantitative, cross-sectional exploratory design, we examined 130 students (106 girls and 24 boys) aged 11 to 16 years (mean age = 12.9, SD = 1.35) from 6th to 8th grade. Twenty-eight participants met the criteria for PTSD, 15 met the criteria for DEP, 43 met the criteria for both PTSD+/DEP+, and 44 served as the control group. Comparative analyses were conducted using the MANOVA and multiple one-way ANOVA tests. **Results**: The MANOVA test indicated an interaction between cognitive and sleep alterations. Post hoc analysis revealed that sleep patterns were significantly altered among the groups with PTSD, DEP, and PTSD+/DEP+ (F(3, 126) = 16.98, *p* = 0.001). In contrast, cognitive alterations were most pronounced in PTSD and PTSD+/DEP+ (F(3, 126) = 63.97, *p* < 0.001). **Conclusions**: These findings emphasize the impact of PTSD and DEP on cognition and sleep. Potential clinical implications suggest the need for interventions targeting sleep and cognitive alterations. This study underscores the complex relationship among traumatic experiences, depression, and cognitive/sleep alterations.

## 1. Introduction

Post-traumatic stress disorder (PTSD) is a severe and often long-lasting mental disorder that can develop in children and adolescents after experiencing traumatic events. These events may involve severe harm or the threat of harm to oneself or others, such as violence, accidents, natural disasters, or injuries [1].

It is estimated that most children and adolescents will experience at least one traumatic event before reaching adulthood. Such experiences can impact their interactions with peers and affect their academic performance, creating additional stress for both the children and their families [2,3]. Furthermore, these traumatic events can increase the likelihood of developing mental health issues in adulthood [1,4]

Despite efforts to protect this population, concerns continue to grow. According to UNICEF [5], 650 million girls and women, as well as 450 million boys and men, have been victims of childhood sexual abuse. Additionally, approximately 468 million children live in conflict zones and are often recruited into these conflicts [6]

While this is a global issue, the situation is particularly severe in certain countries. For instance, in Colombia, the National Institute of Legal Medicine and Forensic Sciences reported in 2023 that violent incidents involving children and adolescents increased by nearly 30% compared to previous years [7].

Despite the high rates of trauma exposure, only a minority of affected children develop post-traumatic stress disorder (PTSD) [8]. However, PTSD is not the only possible outcome; early adversity has also been linked to a higher risk of developing depression in this population [9,10]. While PTSD and depression are distinct diagnoses, they often occur together [11], indicating that shared factors may contribute to both disorders. These factors may include cognitive alterations [10,12] and sleep disturbances [13,14], which can exacerbate the negative effects of trauma on academic and social success among children and adolescents [10,14].

In terms of cognitive alterations associated with trauma experiences, traditional approaches have primarily focused on the content of thoughts related to traumatic experiences, which supports many talking therapies [15]. However, emerging evidence indicates that cognitive processes—such as attention, memory, and executive functions—can also be affected by trauma [10,12]. Specifically, traumatic responses may impair inhibitory control in children and adolescents, a crucial component of executive functions [10,12,16].

Recent research by Calderon-Delgado et al. has utilized fMRI to demonstrate that children with PTSD exhibit inhibitory control problems linked to reduced activation in the frontal lobes, one of the last brain structures to mature fully [17]. Inhibitory control is vital for regulating emotions, especially negative emotions stemming from trauma [16,17]. This alteration can impact the body’s ability to manage physiological processes, such as sleep, in a top-down manner, ultimately affecting an individual’s ability to cope with negative emotions [18].

The relationship between coping and sleep has gained attention in recent studies, which show that sleep is essential for emotional regulation through a bottom-up process [19]. A good night’s sleep is critical for both mental and physical health. Conversely, the ability to regulate emotions is important for reducing the negative effects of emotional stress on sleep quality and overall physiology [20,21].

We suggest that this interplay between top-down and bottom-up processes may be a significant factor in both the initial response to trauma and its persistence, particularly in children and adolescents.

Notably, the DSM-5-TR [22] includes sleep disturbances as a criterion for both PTSD and depression. Studies have shown that a history of childhood trauma is associated with poor sleep quality [18,23], independent of diagnostic criteria. Furthermore, retrospective studies suggest that sleep disturbances may be a significant factor linking childhood maltreatment to adverse mental health outcomes in adulthood [24].

Understanding the connection between sleep quality and cognitive alterations with trauma-related disorders, such as PTSD and depression, in children and adolescents may have significant clinical and social implications. Poor sleep quality is not merely a symptom; it can also exacerbate the severity of PTSD and depression, making treatment more challenging and hindering recovery [23,24,25]. By identifying sleep disturbances in traumatized youth early on, targeted interventions can be implemented to improve overall mental health outcomes and potentially prevent the development of chronic conditions. Moreover, changes in sleep patterns may help explain the cognitive alterations seen in children and adolescents who have experienced trauma.

Few studies have specifically explored the relationship between the severity of PTSD and depressive symptoms related to traumatic experiences and the extent of sleep disturbances in children and adolescents, particularly within the maltreatment literature (for a comprehensive review, see Schønning et al. [26]). However, to date, no studies have investigated the potential association between sleep disturbances and cognitive changes in this population. This study hypothesizes that there is a direct relationship between higher sleep alterations and higher cognitive alterations, particularly in children and adolescents affected by depression and/or PTSD symptomatology.

## 2. Materials and Methods

### 2.1. Participants

After receiving approval from the school principals, participants were recruited from a public school in Medellín, Colombia. The study was explained to the students, and informed consent was obtained from their parents, along with informed assent from the students who agreed to participate. Participants were excluded if they had a previous diagnosis of psychopathology or cognitive impairment. Initially, 142 children and their families agreed to take part in the study. However, three participants had a diagnosis of autism spectrum disorder, four had a diagnosis of ADHD, and five had a diagnosis of cognitive impairment. Ultimately, 130 students (106 girls and 24 boys), aged between 11 and 16 and in the 6th to 8th grades, volunteered for the study.

### 2.2. Measures

#### 2.2.1. Children’s Depression Inventory (CDI)

The CDI (Children’s Depression Inventory) is a scale adapted from the Beck Depression Inventory, designed for children and adolescents aged 7 to 17. It assesses the level of depression through self-reported responses and consists of 27 items. Each item is rated on a scale from 0 to 2 based on the severity of symptoms. The total score can range from 0 to 54, with a higher score indicating a more severe level of depression. According to the manual, a cut-off score of greater than 19 is used to categorize depression, and the Colombian validation indicated its Cronbach α score is 0.79 [27,28].

#### 2.2.2. Neurocognitive Symptomatology Associated with Child Trauma Based on DSM-5 (NeuroTrauma-DSM5)

The NeuroTrauma-DSM5 is a 37-item self-report questionnaire explicitly developed to assess the severity of trauma experiences in Colombian children [29]. It is based on the DSM-5 criteria diagnosis and includes an index for cognitive alterations. The first six items relate to the traumatic experiences in a yes/no response format. The following 20 items are based on the DSM-5 criteria; the following seven items relate to cognitive symptoms. Finally, four items related to dissociative and depersonalization symptomatology were included. These 31 items were rated in terms of the symptom frequency on a 4-point Likert scale (e.g., 0 = not at all, 2 = once or less a week, 3 = twice to four a week, 4 = five or more a week). The cut-off point for identifying cognitive symptoms was set at 10.32 (SD 2.28), and the Cronbach α is 0.97.

#### 2.2.3. The Pittsburgh Sleep Quality Index (PSQI)

The PSQI is an 18-item self-report questionnaire that assesses overall sleep quality over a one-month period [30]. It includes items on sleeping habits, sleep disturbances, and daytime impairments, all rated on a 4-point Likert scale. The 18 items generate a total score and sub-component scores for subjective sleep quality, sleep latency, sleep duration, habitual sleep efficiency, sleep disturbances, use of sleeping medication, and daytime dysfunction. For this study, we applied the Colombian validation of this scale with a Cronbach α of 0.71 [31].

### 2.3. Procedure

A clinical psychologist interviewed each individual after obtaining informed consent and assent from 142 participants. Following these interviews, 12 participants were discharged due to mental health issues unrelated to any traumatic experiences, as was described in the participants section. With 130 participants left, the research team met them in small groups (10–12 participants) at the school library with enough space to respond without interference. The three questionaries were applied individually, explaining each one and giving examples when they were appropriate following the application instructions. Based on the NeuroTrauma-DSM5 and the CDI results, we conformed into four groups of participants. The PTSD+/DEP– with PTSD (according to the DSM-5 criteria) without symptoms associated with depression (scores under 19); DEP+/PTSD– with symptomatology associated with depression and without PTSD; PTSD+/DEP+ with PTSD and symptoms associated with depression, and CONTROLS with no symptoms associated with PTSD nor depression. Table 1 contains the demographic characteristics of each group. We compared the PSQI and the cognitive index results among the four groups based on this distribution. Notice that age and scholar level were compared using ANOVA to ensure all groups were equivalent in these demographic variables. In regard to gender, a significant difference was observed among the groups. Additionally, it included the total score from NeuroTrauma-DSM5 and CDI scales for each group.

### 2.4. Data Analysis

The study data were analyzed using JASP version 0.18.3. We visually checked for normality using QQ plots and the Levine test to ensure the variance was consistent across all four groups. Clinical data were analyzed using a MANOVA test to establish the interaction between cognitive and total sleep scores, followed by post hoc analysis using individual one-way analysis of variance (ANOVA) on each dependent variable. Furthermore, a one-way ANOVA was conducted to establish the impact of PTSD and depression on seven different sleep domains and the overall sleep score. We included Cohen d and partial Eta square to determine the effect size. If the ANOVA test showed significant differences, we conducted the Tukey honest significance difference post hoc test for multiple pairwise comparisons.

## 3. Results

To conduct the MANOVA test, we considered PTSD and depression outcomes as the independent variables and cognition and sleep scores as the dependent variables. This analysis revealed significant differences among the groups in both total sleep scores and cognitive scores, with F(Pillai) = 24.147, *p* < 0.001. This suggests an interaction between sleep and cognitive alterations. For post hoc analysis, individual one-way ANOVAs were conducted for each dependent variable. The results indicated that sleep patterns were significantly altered among the groups with PTSD, DEP, and the combined group (PTSD+/DEP+) (F(3, 126) = 16.98, *p* = 0.001, *η_p_*^2^ = 0.288). In contrast, cognitive alterations were particularly pronounced in the PTSD group and the combined PTSD+/DEP+ group (F(3, 126) = 63.97, *p* < 0.001, *η_p_*^2^ = 0.604).

Additionally, the ANOVA test indicated significant differences among the groups across various sleep domains, with small to moderate effects (according to the partial Eta Square). Specifically, the differences appeared in sleep quality (F(3, 126) = 7.793, *p* < 0.001, *η_p_*^2^ = 0.157), sleep latency (F(3, 126) = 6.698, *p* < 0.001, *η_p_*^2^ = 0.138), sleep duration (F(3, 126) = 3.298, *p* = 0.02, *η_p_*^2^ = 0.073), sleep disturbance (F(3, 126) = 10.246, *p* < 0.001, *η_p_*^2^ = 0.196), and daytime dysfunction (F(3, 126) = 12.58, *p* < 0.001, *η_p_*^2^ = 0.23), and total score (F(3, 126) = 16.987, *p* < 0.001, *η_p_*^2^ = 0.288) (see Table 2).

**Figure 1:**
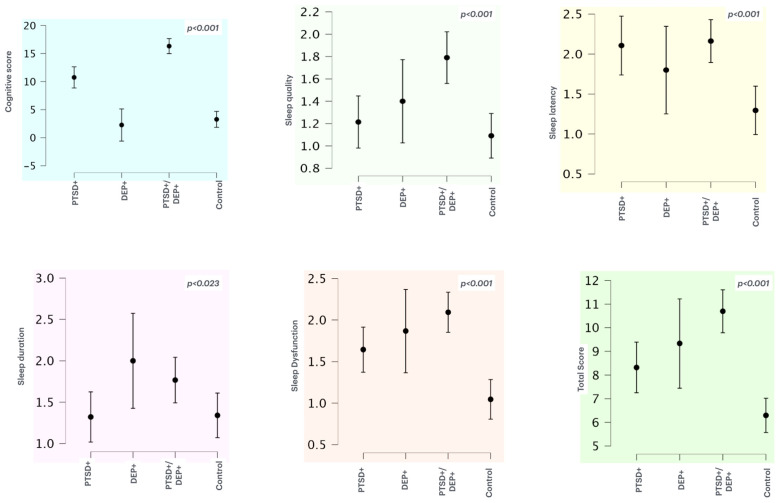
Comparison of cognitive score and those sleep variables that exhibited significant differences among the four groups.

Figure 1 compares the mean and standard deviation of the total cognitive score and the sleep variables that showed significant differences (*p* < 0.005) among the four groups.

Table 3 presents the results of the Tukey post hoc test, highlighting specific differences between the groups for various domains. The most significant differences were found when comparing the PTSD+/DEP+ group with the CONTROL group, particularly in sleep quality (*p* < 0.001), sleep latency (*p* = 0.001), sleep disturbance (*p* < 0.001), daytime dysfunction (*p* < 0.001), and the total score (*p* < 0.001). The PTSD+/DEP+ group exhibited greater alterations in these areas.

Furthermore, notable differences were observed when comparing the PTSD+ group with the CONTROL group in sleep latency (*p* = 0.005), daytime dysfunction (*p* = 0.015), and the total score (*p* = 0.024), again showing higher alterations in the PTSD+ group.

Lastly, differences were noted when comparing the DEP+ group with the CONTROL group in daytime dysfunction (*p* = 0.005) and the total score (*p* = 0.004), with the DEP+ group demonstrating higher alterations in these metrics.

## 4. Discussion

The current study aimed to identify the relationship among cognitive alterations, sleep quality, and symptoms of PTSD and/or depression following a traumatic experience in a sample of children. To the best of our knowledge, this is the first study to report the association of PTSD and depression symptoms with cognitive and sleep disturbances in a Colombian sample of children and adolescents.

The findings indicate that cognitive alterations are more closely related to PTSD than to depressive symptoms. The cognitive complaints observed include difficulties in learning, sustained attention, emotional regulation, and inhibitory control. Previous studies have established a relationship between cognitive alterations and PTSD through neuropsychological assessments. Notably, most of these complaints are associated with the frontal lobes, which are among the last brain structures to fully develop.

Emotional regulation and inhibitory control are closely linked among various cognitive complaints [32,33]. Regulating emotions involves the ability to inhibit certain aspects of emotional responses. As children’s frontal lobes mature, they develop the capacity to manage their emotional states in a top-down manner [34]. This maturation process is influenced by neurodevelopment and external experiences, such as parenting styles, education, and cultural norms [34]. Traumatic experiences can disrupt this developmental process, impairing children’s ability to effectively manage their emotions [35,36].

In regard to sleep disturbance, the outcomes suggest that they varied by mental health condition, being worse in those children affected by depression and PTSD simultaneously. In this group, sleep quality, sleep latency, sleep disturbance, daytime dysfunction, and total score were the variables more affected, suggesting that sleep alterations, as a whole, are crucial variables to consider in the assessment and intervention process with this population. Regarding the association of early traumatic experiences with sleep alterations, the outcomes of the study support the growing evidence implicating sleep as another dimension of health consequences from child adversity [37,38].

Sleep is also closely associated with emotional regulation, sharing brain structures, and neurochemical activities. Almost all affective disorders coexist with sleep abnormalities, leading researchers like Palmer et al. to suggest a strong relationship between these two areas [25]. While opinions differ regarding the exact function of sleep, it is widely accepted that sleep plays an adaptive role in processing stressors and emotions [39,40]. Sleep difficulties, such as those experienced by the group with PTSD and depression, can impact the ability to cope with negative emotions. Since sleep is primarily a physiological process, it is influenced by various bodily signals regulated by autonomic activity [40,41]. Similarly, the body’s response to traumatic events also involves autonomic activity, which can trigger a fight-or-flight response [36]. In our view, sleep disturbances reflect the intensity of autonomic activity resulting from traumatic experiences, which affects individuals’ ability to cope with the emotions associated with that trauma in a bottom-up manner.

We believe that the interaction between top-down cognitive processes and bottom-up physiological responses plays a crucial role in determining how individuals react to traumatic events. This perspective may help explain the significant variability in children’s responses following such events. Some children may exhibit heightened physiological reactivity and experience sleep difficulties, while others may show more cognitive alterations. Given that the evidence for identifying the most effective treatments for children and adolescents with PTSD is considerably less comprehensive than that for adults [42], this viewpoint could promote the development of tailored and innovative assessment and intervention strategies aimed at reducing the impact of trauma in this population.

This study did not intend to establish a causal/effect relationship. Instead, it intended to describe the association between depression and PTSD with cognitive and sleep alterations to underscore the necessity of taking cognitive and sleep disturbances into account as a modulator of treatment effectivity. The outcomes of this study suggest a cause-effect relationship between sleep and cognition. Further studies are necessary to clarify this potential relationship. Nevertheless, improving sleep quality and including cognitive training could enhance social and academic outcomes that positively impact children and adolescents’ mental health.

Our research has several limitations. First, the sample does not represent the broader Colombian population. Future research should evaluate sleep disturbances in more diverse clinical and non-clinical contexts. Second, there are other important factors that can influence sleep, such as socioeconomic status, housing conditions, neighborhood environment, and parental stability. To our knowledge, this is the first study to explore the relationship between sleep and cognition in children who have experienced trauma. The findings highlight the complex interplay among traumatic experiences, depression, and alterations in cognitive and sleep functions, indicating a need for further research in this area.

## Figures and Tables

**Table 1:** Sociodemographic data for the four groups. Means, standard deviation, percentages, and *p*-value.

Variable	PTSD+ *n* = 28	DEP+ *n* = 15	PTSD+/DEP+ *n* = 43	CONTROLS *n* = 44	*p*-Value (Levene Test)
Age	13.3 ± 1.5	12.7 ± 1.4	13 ± 0.9	12.9 ± 1.6	0.480 (0.045)
Educational level	7.1 ± 0.8	7.1 ± 0.9	7.2 ± 0.7	6.7 ± 0.9	0.06 (0.057)
Gender (%)					0.015
Girls	78.6	93.3	93	68.2	
Boys	21.4	6.7	7	31.9	
PTSD	25.35 ± 13.01	3.46 ± 9.84	34.67 ± 9.84	5.22 ± 7.71	<0.001 (0.036)
Depression	13.21 ± 3.37	23.8 ± 4.41	26.74 ± 5.52	11.15 ± 3.38	<0.001 (0.004)

ANOVA one way for numerical data and X^2^ for nominal data. PTSD+ = Participants with PTSD; DEP+ = Participants with depression.

**Table 2:** Comparison of sleep domains with PTSD and depression as organizing diagnosis.

Variable	Groups	Mean ± SD	F (3, 126)	*p*	*η_p_* ^2^
Cognitive score	PTSD+	11.89 ± 5.71	63.97	<0.001	0.604
	DEP+	2.46 ± 6.37			
	PTSD+/DEP+	17.74 ± 4.66			
	CONTROLS	3.5 ± 5.22			
Sleep quality	PTSD+	1.21 ± 0.63	7.793	<0.001	0.157
	DEP+	1.4 ± 0.73			
	PTSD+/DEP+	1.79 ± 0.77			
	CONTROLS	1.09 ± 0.67			
Sleep latency	PTSD+	2.1 ± 0.99	6.698	<0.001	0.138
	DEP+	1.8 ± 1.08			
	PTSD+/DEP+	2.16 ± 0.89			
	CONTROLS	1.29 ± 1.02			
Sleep duration	PTSD+	1.32 ± 0.81	3.298	0.023	0.073
	DEP+	2.0 ± 1.13			
	PTSD+/DEP+	1.76 ± 0.92			
	CONTROLS	1.34 ± 0.92			
Sleep efficiency	PTSD+	0.21 ± 0.63	2.335	0.077	0.053
	DEP+	0.4 ± 0.91			
	PTSD+/DEP+	0.41 ± 0.73			
	CONTROLS	0.09 ± 0.29			
Sleep disturbance	PTSD+	1.6 ± 0.62	10.24	0.196	0.196
	DEP+	1.73 ± 0.79			
	PTSD+/DEP+	2.07 ± 0.59			
	CONTROLS	1.34 ± 0.56			
Sleep medication	PTSD+	0.21 ± 0.63	1.964	0.0123	0.045
	DEP+	0.13 ± 0.51			
	PTSD+/DEP+	0.39 ± 0.82			
	CONTROLS	0.09 ± 0.29			
Daytime dysfunction	PTSD+	1.64 ± 0.73	12.580	<0.001	0.230
	DEP+	1.86 ± 0.99			
	PTSD+/DEP+	2.09 ± 0.81			
	CONTROLS	1.04 ± 0.8			
Total Score	PTSD+	8.32 ± 2.88	16.987	<0.001	0.288
	DEP+	9.33 ± 3,73			
	PTSD+/DEP+	10.69 ± 3.05			
	CONTROLS	6.29 ± 2.45			

*η_p_*^2^: Partial Eta Square.

**Table 3:** Tukey post hoc test findings.

PSQI	*p*	*η_p_* ^2^	PTSD+ DEP+	PTSD+ PTSD+/DEP+	PTSD+ Controls	DEP+ PTSD+/DEP+	DEP+ Controls	PTSD+/DEP+ Controls
Sleep quality	<0.001	0.157	0.845	0.006 **	0.888	0.259	0.464	<0.001 ***
Sleep latency	<0.001	0.138	0.764	0.996	0.005 **	0.610	0.321	0.001 ***
Sleep duration	0.023	0.073	0.105	0.198	1.000	0.836	0.085	0.143
Sleep disturbance	<0.001	0.196	0.920	0.013 *	0.288	0.272	0.152	<0.001 ***
Daytime dysfunction	<0.001	0.230	0.826	0.110	0.015 *	0.791	0.005	<0.001 ***
Total Score	<0.001	0.288	0.699	0.006 **	0.024 *	0.404	0.004 **	<0.001 ***

* *p* < 0.05; ** *p* < 0.01; *** *p* < 0.001.

## Data Availability

Data for this study are not publicly available due to ethical reasons.
